# Characteristics of premonitory urge and tic symptom in different age groups from a network perspective

**DOI:** 10.3389/fpsyt.2025.1530911

**Published:** 2025-06-03

**Authors:** Liping Yu, Yanlin Li, Xianbin Wang, Yonghua Cui

**Affiliations:** ^1^ Department of Psychiatry, Beijing Children’s Hospital, Capital Medical University, National Center for Children’s Health, Beijing, China; ^2^ Peking University Sixth Hospital, Peking University Institute of Mental Health, National Health Commission (NHC) Key Laboratory of Mental Health (Peking University), National Clinical Research Centre for Mental Disorders (Peking University Sixth Hospital), Beijing, China

**Keywords:** tic disorders, premonitory urges, age, network analysis, behavioral therapy

## Abstract

**Background:**

Tic disorders are neurodevelopmental disorders. Current research suggests that premonitory urges (PU) may be the cause and basis of tic disorders. The study used network analysis to investigate the detailed associations between PUs and tic symptoms the and internal relationships of PUs.

**Methods:**

We included 1021 outpatient tic disorders. We used R 4.1.3 to perform network analysis. The “qgraph” package constructed the Premonitory Urge for Tics Scale (PUTS) and Yale Global Tic Severity Scale (YGTSS)networks for low-age and high-age group, and calculated the centrality measures for the four networks. We used the “bootnet” package to test the accuracy of the four network models and used the “NetworkComparisonTest” package to compare the networks for different age groups.

**Results:**

The network structures were stable (above 0.5). The node with the highest intensity was the 8th entry. The “connection” node was identified as the “all defects caused by motion or vocal tics” entry in the YGTSS. The strength and weight of the network structures did not significantly differ between the different groups. The node strength substitution test revealed that the 9th item had a significantly stronger intensity in lower age group (0.75) than in the higher age group (0.53) with a *P*-value of 0.03. The strength in the network was also significantly greater in the younger age group (1.34) than in the older age group (1.02) with a *P*-value of 0.04.

**Conclusion:**

This study validates the internal structure of the PUTS scale across different age groups from a network perspective. It provides theoretical guidance and practical basis for targeted behavioral therapy.

## 1 Introduction

Tic disorders (TDs) are neurodevelopmental disorder that typically begins in childhood or adolescence (1). They are characterized by sudden, rapid, non-rhythmic, repetitive movements and/or vocalizations involving the head, face, limbs, and trunk (2). TD can be broadly categorized into transient tic disorder, chronic motor or vocal tic disorder, and Tourette syndrome (TS) (3). In China, the prevalence of tic disorders is relatively high, with an overall incidence exceeding 3% (4).

Premonitory urge (PU) refers to a sensory phenomenon occurring before the onset of tic symptoms, such as itching, pressure, or a sense of incompleteness (5). Tics are seen as motor or vocal responses that can alleviate the intensity of PU a way of relieving discomfort through the manifestation of motor or vocal tics (6). For example, a child with a tic such as “eye blinking” may describe the act as a response to the sensation of itching in the eyes, finding relief through the blinking movement. Therefore, PU serves as an internal process and a critical foundation for tics, potentially contributing to their origin. Importantly, PU plays an important role in behavioral therapy for tic disorders, as seen in widely used approaches such as habit reversal training, where the first step is to recognize PU before tics occur (7).

Currently, research into TD and PU often relies on the total scores or factor scores from psychological assessment scales. This approach treats tic symptoms or abnormal sensory phenomena as outcomes influenced by underlying common factors, interpreting the severity of symptoms through total scores and overlooking the interactions between symptoms and features. Traditional research methods often employ analyses such as correlation and regression, using the total score scales as the unit of analysis, and neglecting the “connecting points” where overall features and PU influence specific symptoms of motor and vocal tics.

In 2008, Borsboom proposed the network theory of psychopathology (8). The main idea behind psychopathological networks is that psychological networks can be described as dynamically interacting topological structures, where strong connections within the network are more likely to develop into mental disorders. Certain nodes have stronger connections, and activating these nodes can more easily stimulate the whole network (9). The network theory of psychopathology is based on network analysis, which is derived from graph theory in mathematical theory. The construction of a network model involves two elements: nodes and edges. Network analysis can visualize the complex relationships between internal nodes. Additionally, centrality indices in network analysis include strength, closeness, and betweenness. Strength refers to the sum of the absolute weighted correlations of all connections of a node relative to all other nodes. Closeness is the inverse of the sum of the distances from a given node to all other nodes. A higher closeness of a node indicates shorter average distances to all nodes, and vice versa. Betweenness is the degree to which a node lies between other nodes, calculated as the number of times a node lies on the shortest path between two other nodes. The higher the betweenness of a node, the more it lies between other nodes. In psychopathological networks, nodes represent different symptoms, edges represent the correlations between symptoms, and edge weights reflect the strength of the correlation between two nodes. The centrality indices of nodes reflect the status of symptoms in the network, indicating their importance in the disease (10). As of 2018, 204 studies are using psychopathological network analysis in clinical practice (9). This emerging theory has matured over the past decade, with numerous studies finding value in psychopathological network analysis for inferring causal relationships between symptoms and identifying core symptoms, providing new perspectives and directions for the study of mental and psychological disorders.

Therefore, this study aims to utilize cross-sectional data collected during the first visits to the psychiatric department of Beijing Children’s Hospital to establish a network model. The aim is to explore the network structure and core features of PU and tic symptoms in children with TD. Based on literature reviews and preliminary investigations, we will focus on the detailed item network structures of the PUTS and YGTSS scales for children with TD in different age groups. We chose to use network analysis because it can reveal the internal relationships between tic symptoms that traditional methods are unable to capture, as well as the associations between tic symptoms and PU. By analyzing the PU-YGTSS network across different age groups, we can further explore the impact of age on symptom associations. Using network analysis techniques, we will further explore the “connecting points” where PU influence tics, providing data support for an in-depth exploration of the specific relationship between PU and tics and identifying precise targets for behavioral interventions.

## 2 Methods

### 2.1 Study participants and data collection

Clinical assessment data of children with TD who visited the tic outpatient clinic at the psychiatric department of Beijing Children’s Hospital from June 2021 to October 2022 were retrospectively reviewed for analysis. The inclusion criteria were as follows (1): children included in the study were screened by psychiatric physicians, met the criteria for TD (according to the Fifth Edition of the Diagnostic and Statistical Manual of Mental Disorders, DSM-5), and had not received medication; (2) age between 6 and 12 years; (3) Cooperative ability during testing. Exclusion criteria included: (1) any other current major psychiatric disorder except for obsessive-compulsive disorder (OCD) and attention deficit hyperactivity disorder (ADHD); (2) Comorbid severe somatic or neurological illness (e.g., epilepsy, traumatic brain injury).

**Figure 1:**
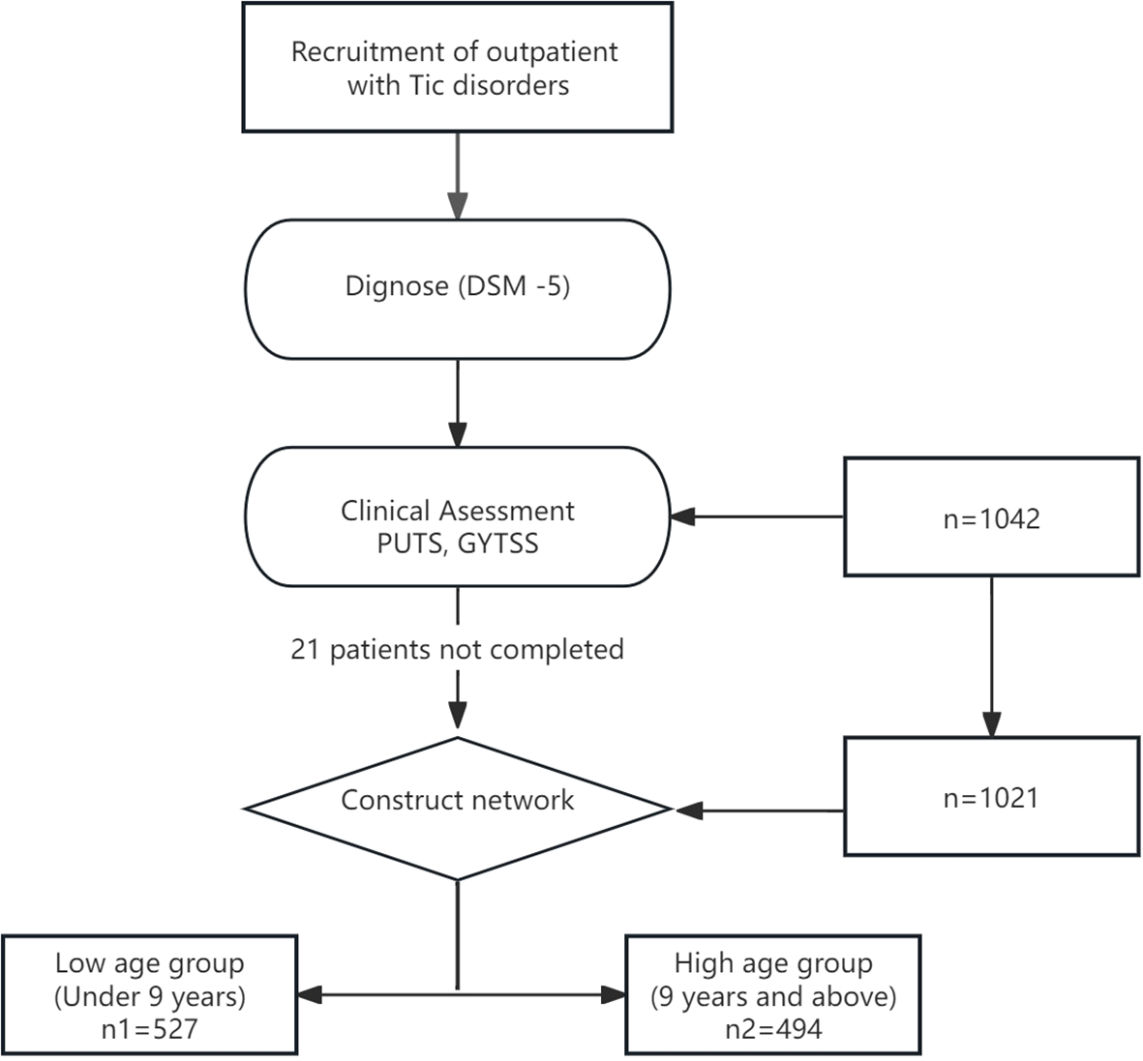
The flow diagram for the identification data for the study.

The psychological assessment tests consisted of three parts: a basic information form, self-report scales, and observer-rated scales. The basic information form and self-report scales were completed by the patients themselves or their parents using a mobile app, while the observer-rated scales were administered by professional psychological evaluators using the Children’s Psychological Assessment System. A total of 1042 children and adolescents with tic disorders participated in this study, with 21 individuals failing to complete the entire survey and subsequently being excluded. Finally, 1021 cases of children aged 6–12 years were included. Based on the median age, those under 9 years (exclusive) were defined as the low age group, and those aged 9 years and older were defined as the high age group. For more details, see **Figure 1**.

This study was approved by the Ethics Committee of Beijing Children’s Hospital (No. 2023-E-105-R)., Capital Medical University, and informed consent was obtained from the participants and their guardians.

### 2.2 Assessment tools

#### 2.2.1 Premonitory urge for tics scale

The PUTS is a tool developed by Professor Woods in 2005 to assess PU (11). Widely employed in tic-related clinical research, the scale has been translated into various languages and is the most widely used tool, exhibiting excellent psychometric properties. The PUTS reports on the sensations and severity associated with tics but does not include the location of PU. Scoring is as follows: None: 1 point; Some: 2 points; Quite a few: 3 points; A lot: 4 points. The total score ranges between 9 and 36. The reliability and validity of this scale have been confirmed in the Chinese population (12).

#### 2.2.2 YGTSS

Tic severity was measured by the Yale Global Tic Severity Scale (YGTSS) (Leckman et al., 1989). The YGTSS is a semi-structured interview designed to assess the nature and severity of motor tics (ranged 0-25) and vocal tics (ranged 0-25), which was the sum of the motor and vocal tic scores (0–50). In addition, it also includes an impairment scale, which is scored from 0 to 50. Thus, the total YGTSS score was ranged from 0 to 100. A higher score indicates a more severe condition. We used the validated Chinese version of YGTSS (13, 14), performed by trained psychologists.

### 2.3 Network analysis

#### 2.3.1 Construction of network models

The “graph” package in R (version 4.1.3, The University of Auckland, Auckland, New Zealand) was used to construct network models and calculate centrality indices. Each scale item was represented as a node and the edges between nodes represented associations in the construction of network model. In a partial network model, the absence of an edge between two nodes indicates a conditionally independent relationship between the nodes. Edge strength or weight can be understood as an estimate of partial correlation coefficients, representing the correlation between two nodes when controlling for all other nodes in the network. We constructed Gaussian graphical models (GLASSO networks) for the PUTS in the low age group (below 9 years) and high age group (9 years and above) to explore the internal relationships of premonitory urges. We also constructed GLASSO networks for the PUTS-YGTSS in the low age and high age groups to examine the relationships between premonitory urges and motor tics, vocal tics, and tic impairment. The GLASSO algorithm was selected for its ability to handle high-dimensional data and produce sparse networks by applying regularization (λ = 0.5), which removes weak correlations (|r| < 0.15) and highlights robust connections. This approach enhances interpretability and reduces noise, making it suitable for identifying clinically relevant symptom interactions. We use regularization selection as a parameter for network construction, which helps improve the reproducibility and transparency of the research.

In order to present visual results clearly, the “layout” parameter controlled the layout of the graph, and the “spring” layout was chosen to control the positions of the node. The “spring” layout utilized the Fruchterman-Reingold algorithm to form an embedded matrix, where nodes (whether connected or not) repelled each other and connected nodes attracted each other. After several iterations (default is 500), during which the maximum displacement of each node decreased, the layout achieved a correspondence between the distances among nodes and the absolute edge weights. In other words, symptoms with lower strength and fewer connections were placed further apart, while symptoms with more connections or stronger correlations were placed closer together. Given the large size of our dataset and the interactions between variables, we used the graphical lasso algorithm (graph = “glasso”) with regularization to estimate the precision matrix. This approach considered potential structures and sparsity between variables, removing some intricate weak correlations, resulting in a simplified and tidy network model for easier identification of key information. The ‘palette’ parameter adjusted the color of the network between PUTS items and YGTSS items, using ‘pastel’ for the low age group and ‘colourblind’ for the high-age group for better differentiation. The “minimum” parameter was set to 0.15 to hide edges with absolute weights below 0.15, and the “negDashed” parameter was set to “TRUE”, while other parameters remained default, indicating that edges showing positive correlations were displayed as green solid lines and negative correlations as red dashed lines. The thickness of the edges represented the strength of the association, with thicker lines indicating stronger associations. Finally, the “filetype” parameter was used to save the visualization image.

Centrality indices, including strength, closeness, and betweenness (15), were calculated using the “centralityPlot” function. Strength represents the sum of the absolute weighted correlation coefficients of all connections of a node relative to all other nodes. Closeness is the reciprocal of the sum of the distances from a givin node to all other nodes. A higher closeness indicates a shorter average distance to all nodes, and vice versa. Betweenness measures the extent to which a node falls between other nodes, calculated as the number of times a node lies on the shortest path between two other nodes. The higher the betweenness, the more the node acts as an “intermediary”. The ‘scale’ parameter was set to z-scores, indicating the use of standard scores for centrality indices.

The “bootnet” package was employed to assess the accuracy of the network analysis. We calculated d the stability of centrality indices for nodes. The stability of centrality indices was measured using correlation stability coefficients (CS values), with CS values generally recommended to be above 0.25 and preferably greater than 0.5 (16). The calculation was based on the “case-dropping subset” bootstrap method using the “corStability” function.

The “bootnet” package was also used to calculate confidence intervals. The number of bootstraps was set to 1000, and the number of cores for parallel computing was set to 5. Based on these results, differences in strength, closeness, and betweenness for nodes could be calculated.

#### 2.3.2 Network model comparison

The comparison test was conducted using the “NetworkComparisonTest” package. We compared whether there were significant differences in edge weights and overall strength between the low and high age groups in the PUTS network. Similarly, we compared whether there were significant differences in edge weights, overall strength, edge weights in permutation tests, and overall strength in permutation tests between the low and high age groups in the PUTS-YGTSS network. The purpose of this comparison was to determine whether there were differences in the networks between the low and high age groups.

## 3 Results

### 3.1 Descriptive statistics

A total of 1021 children with tic disorders fully participated in this study, all of whom were from the psychiatric outpatient department of Beijing Children’s Hospital. The age of the participants ranged from 6 to 12 years, with 527 individuals in the low age group and 494 individuals in the high age group, making the two groups approximately equal in size with a ratio of about 1:1. See **Table 1**.

**Table 1:** Age groups included in the network analysis.

	Categories	sample size (n = 1021)	Percentage(%)
Age group	low age group	527	51.6
	High age group	494	48.4

**Figure 2:**
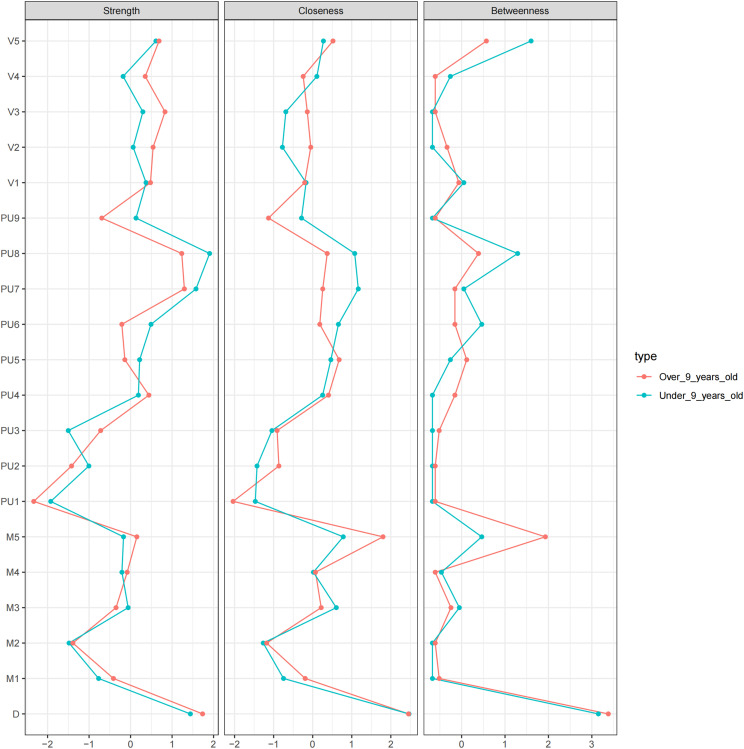
The PUTS-YGTSS network.

**Figure 2** shows the correlation network model between the PUTS and YGTSS scales. The network is grouped into four clusters: PUTS, YGTSS_D (total impairment due to motor and/or vocal tics), YGTSS_M (motor tics), and YGTSS_V (vocal tics), each represented by a different color. The graph shows that PU8 and D (total impairment due to motor and/or vocal tics) have strong associations with other nodes in both networks. While the overall structures of the networks for the low and high age groups are similar, there are differences in the details. The network for low age group has more edges, but these edges are thinner, indicating weaker association strength. In the low age group, PU8 has the highest strength, D (total impairment due to motor and/or vocal tics) has the highest closeness, and M4 (complexity of motor tics) has the highest betweenness. In the high age group, PU8 has the highest strength and betweenness, while M5 (impact of motor tics) has the highest closeness. More precise and detailed differences are described in the following sections.

A network with only nine nodes of PUTS (PUTS network) was also built. We can see that the thickest line in both models is “PU7-PU8.” “PU1-PU4” shows a strong negative correlation, which is more pronounced in the low age group. (**Appendix Figure S1**)

Note: The green solid line represents positive correlation, the red dotted line represents negative correlation, and the thickness of the edge indicates the strength of the correlation. PU1: Before the tic occurred, I felt an inner itchy sensation, PU2: Before the tic occurred, I felt pressure in my head or body, PU3: Before the tic occurred, I felt inner tension and uneasiness, PU4: Before the tic occurred, I felt uncomfortable or uneasy feelings, PU5: Before the tic occurred, I felt a3sense of something unfinished, PU6: Before the tic occurred, I felt inner “energy”.PU4: Before the twitch, I feel a sense of discomfort or uneasiness, PU5: Before the twitch, I feel a sense of something unfinished, PU6: Before the twitch, I feel a sense of inner “energy” coming out, PU7: These feelings occur frequently before the twitch., PU8: These sensations occur with almost every twitch, PU9: After the onset of the twitch, these itchy sensations, nervousness, stress, or uncomfortable, uncomfortable, unfinished sensations fade away or persist for a short period of time, M1: number of motor tic species, M2: frequency of motor tics, M3:Motor tic intensity, M4: Motor tic complexity, M5: Motor tic interference, V1: Vocal tic species, V2: Vocal tic frequency, V3: Vocal tic intensity, V4: Vocal tic complexity, andV5: vocal tic interference, D: Total deficit due to motor and/or vocal tics

**Figure 3:**
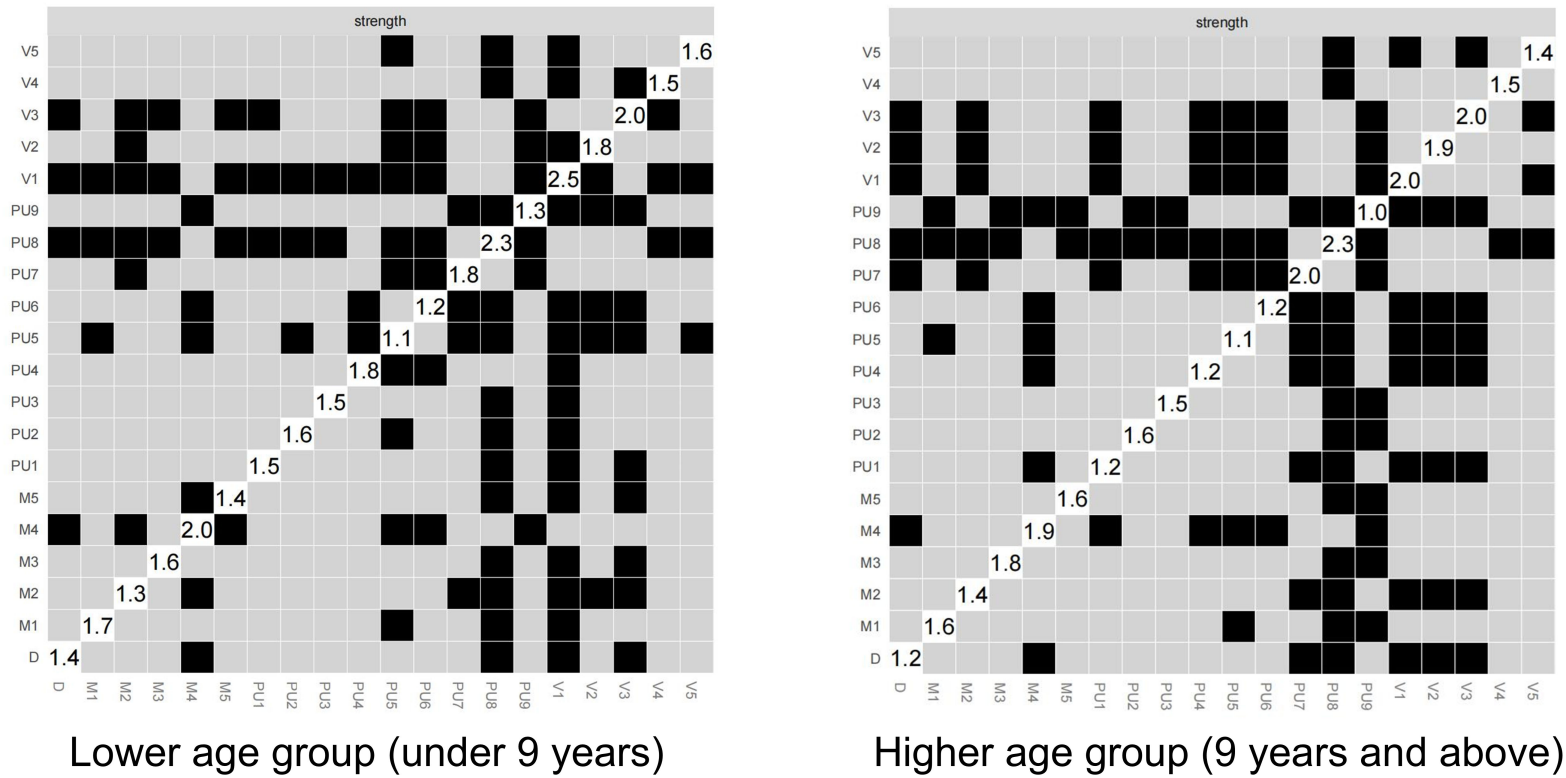
The centrality indicators for the PUTS-YGTSS network.

Note: The green solid line represents positive correlation, the red dotted line represents negative correlation, and the thickness of the edge indicates the strength of the correlation. PU1: Before the tic occurred, I felt an inner itchy sensation, PU2: Before the tic occurred, I felt pressure in my head or body, PU3: Before the tic occurred, I felt inner tension and uneasiness, PU4: Before the tic occurred, I felt uncomfortable or uneasy feelings, PU5: Before the ic occurred, I felt a sense of something unfinished, PU6: Before the tic occurred, I felt inner “energy”.PU4: Before the twitch, I feel a sense of discomfort or uneasiness, PU5: Before the twitch, I feel a sense of something unfinished, PU6: Before the twitch, I feel a sense of inner “energy” coming out, PU7: These feelings occur frequently before the twitch., PU8: These sensations occur with almost every twitch, PU9: After the onset of the twitch, these itchy sensations, nervousness, stress, or uncomfortable, uncomfortable, unfinished sensations fade away or persist for a short period of time, M1: number of motor tic species, M2: frequency of motor tics, M3:Motor tic intensity, M4: Motor tic complexity, M5: Motor tic interference, V1: Vocal tic species, V2: Vocal tic frequency, V3: Vocal tic intensity, V4: Vocal tic complexity, andV5: vocal tic interference, D: Total deficit due to motor and/or vocal tics

**Figure 3** shows the centrality indicators for the PUTS-YGTSS network. The green lines represent the low age group and the red lines represent the high age group. In the low age group, PU8 has the highest strength, D has the highest tightness, and M4 (motor twitch complexity) has the highest degree of mediation. In the high age group, PU8 has the highest strength and degree of mediation, and M5 (motor twitch interference) has the highest tightness. Meanwhile, the centrality indicators for PUTS network indicated that in the low-age group, PU8 (“These sensations occur almost every time I have a tic”) has the highest strength, closeness, and betweenness. Similarly, in the high age group, PU8 has the highest strength, closeness, and betweenness. (**Appendix Figure S2**)

**Figure 4:**
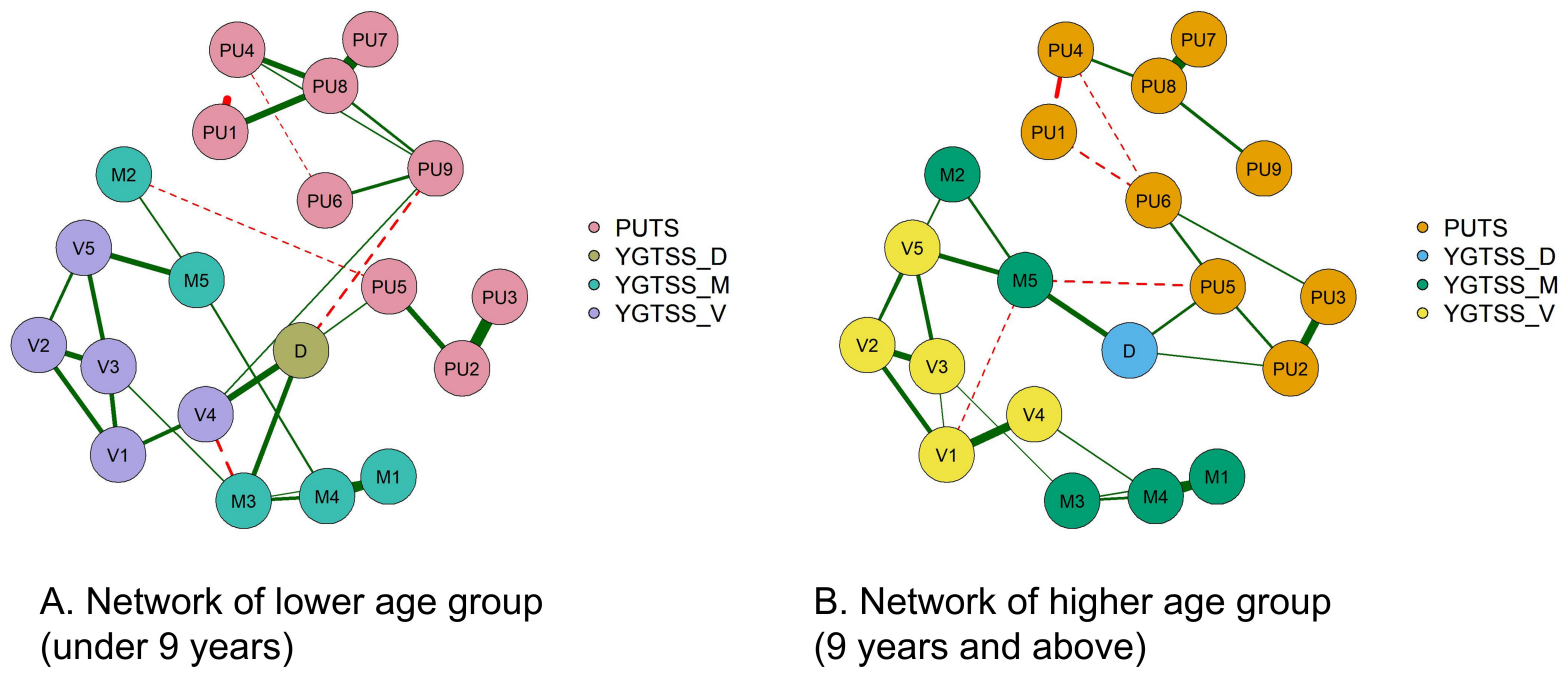
The differential test for PUTS-YGTSS network intensity across different age groups. PU1: Before the tic occurred, I felt an inner itchy sensation, PU2: Before the tic occurred, I felt pressure in my head or body, PU3: Before the tic occurred, I felt inner tension and uneasiness, PU4: Before the tic occurred, I felt uncomfortable or uneasy feelings, PU5: Before the tic occurred, I felt a sense of something unfinished, PU6: Before the tic occurred, I felt inner "energy". PU4: Before the twitch, I feel a sense of discomfort or uneasiness, PU5: Before the twitch, I feel a sense of something unfinished, PU6: Before the twitch, I feel a sense of inner "energy" coming out, PU7: These feelings occur frequently before the twitch. , PU8: These sensations occur with almost every twitch, PU9: After the onset of the twitch, these itchy sensations, nervousness, stress, or uncomfortable, uncomfortable, unfinished sensations fade away or persist for a short period of time, M1: number of motor tic species, M2: frequency of motor tics, M3:Motor tic intensity, M4: Motor tic complexity, M5: Motor tic interference, V1: Vocal tic species, V2 : Vocal tic frequency, V3 : Vocal tic intensity, V4 : Vocal tic complexity, and V5 : vocal tic interference, D: Total deficit due to motor and/or vocal tics.

**Figure 5:**
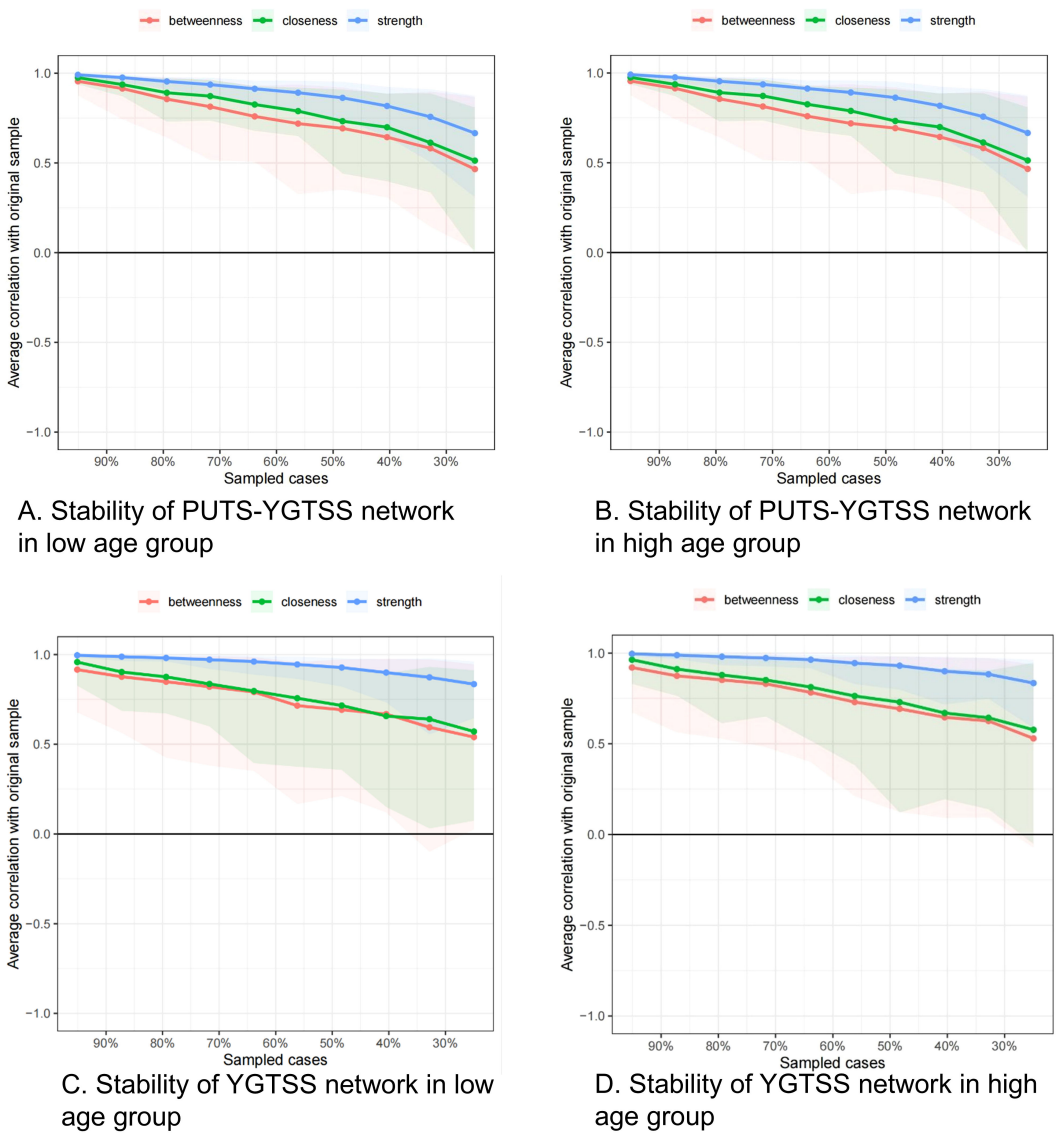
**(A)** stablity of PUTS-YGTSS network in lower age group (under 9 years); **(B)** stablity of PUTS-YGTSS network in higher age group (9 years and above); **(C)** stablity of YGTSS network in lower age group (under 9 years); **(D)** stablity of YGTSS network in higher age group (9 years and above).

**Figures 4** show the stability of the centrality indices for nodes. The blue lines represent strength, the blue filled area represents the confidence interval for strength, the green lines represent closeness, the green filled area represents the confidence interval for closeness, the red lines represent betweenness, and the red filled area represents the confidence interval for betweenness. The stability of the centrality indices is objectively measured by the CS values. For PUTS-YGTSS network of the low age group, the CS value is 0.517 for strength, 0.283 for closeness and 0.128 for betweenness. We can consider this network as stable in strength, relatively stable in closeness, and less stable in betweenness (**Figure 5A**). For the PUTS-YGTSS network of the high age group, the CS value is 0.518 for strength, 0.205 for closeness and 0.128 for betweenness. We can consider this network as stable in strength, less stable in closeness, and less stable in betweenness (**Figure 5B**). For the PUTS network the low age group, the CS value is 0.672 for strength, 0.206 for closeness and 0.049 for betweenness. We can consider this network as stable in terms of strength but less stable in terms of closeness and betweenness (**Figure 5C**). For the PUTS network of the high age group, the CS value is 0.672 for strength, 0.283 for closeness and 0.049 for betweenness. We can consider this network as stable in strength, relatively stable in closeness, and less stable in betweenness (**Figure 5D**). Overall, the strength of these four networks is stable. Therefore, in the following difference tests and model comparisons, we mainly focus on the strength as a centrality index.

**Figure 5** illustrates the results of the differential test for PUTS-YGTSS network intensity across different age groups, where black indicates significant differences and gray indicates non-significant differences. The numerical values in the white areas along the diagonal represent the specific values of intensity. The networks across the different age groups shows overall similarities with some differences in detail. Notably, V1 (number of vocal tics) and PU9 (sensations gradually disappearing or lasting for a short time after the tic occurs) shows significant differences between the two networks. In the PUTS-YGTSS network of the low age group, V1 appears with the highest intensity, while in the PUTS-YGTSS network of the high-age group, PU8 reaches the highest intensity. For the PUTS network, it is evident that the PUTS network of the low age group, PU8 shows significant differences in intensity compared to all other items. In addition, PU5 (feeling that something is incomplete before the tic occurs) shows significant differences. In the PUTS network of the high age group, PU8 shows significant differences in intensity with all items except PU7 (these sensations often occur before the tic). After PU8, PU7 shows the next highest intensity differences, except for PU3 (feeling of internal tension and anxiety before the tic) and other items not including PU8. PU8 emerges as the item with the highest intensity in both networks. (**Appendix Figure S3**)

PU1: Before the tic occurred, I felt an inner itchy sensation, PU2: Before the tic occurred, I felt pressure in my head or body, PU3: Before the tic occurred, I felt inner tension and uneasiness, PU4: Before the tic occurred, I felt uncomfortable or uneasy feelings, PU5: Before the tic occurred, I felt a sense of something unfinished, PU6: Before the tic occurred, I felt inner “energy”.PU4: Before the twitch, I feel a sense of discomfort or uneasiness, PU5: Before the twitch, I feel a sense of something unfinished, PU6: Before the twitch, I feel a sense of inner “energy” coming out, PU7: These feelings occur frequently before the twitch., PU8: These sensations occur with almost every twitch, PU9: After the onset of the twitch, these itchy sensations, nervousness, stress, or uncomfortable, uncomfortable, unfinished sensations fade away or persist for a short period of time, M1: number of motor tic species, M2: frequency of motor tics, M3:Motor tic intensity, M4: Motor tic complexity, M5: Motor tic interference, V1: Vocal tic species, V2: Vocal tic frequency, V3: Vocal tic intensity, V4: Vocal tic complexity, andV5: vocal tic interference, D: Total deficit due to motor and/or vocal tics

The NetworkComparisonTest package was used to perform comparative analyses of networks across different age groups, assessing whether there are significant differences in overall intensity and whether permutation tests on edge weights reveal significant differences.

For the low age group, the overall intensity of the PUTS network is 3.57, while it is 3.24 for the high age group. The P-value between the PUTS networks of different age groups is 0.32, indicating no statistically significant differences. For the PUTS-YGTSS networks, the overall intensity is 8.75 for the low age group and 8.23 for the high age group. The P-value between the PUTS-YGTSS networks of different age groups is 0.48, indicating no statistically significant differences in overall intensity. Permutation tests on the intensity of individual nodes show a significant difference for PU9 in both PUTS networks (*p* = 0.03) and PUTS-YGTSS networks (*p* = 0.04) across different age groups. Further details of the permutation tests on edge weights between PUTS networks of different age groups are provided in the **Appendix Figure S4**.

## 4 Discussion

This study used network analysis to construct the PUTS-YGTSS network structure and the PUTS network structure across different age groups, aiming to explore the internal correlations of the PUTS scale and its relationship with the YGTSS.

The results of the PUTS network analysis showed no significant differences in network structures between the different age groups, indicating that the internal correlations of PUTS in our sample are not influenced by age. This finding supports the application of PUTS in the 6–12 age group. In addition, we observed a negative correlation between PU1 (‘I feel an internal itch before the tic occurs’) and PU4 (‘I feel uncomfortable or uneasy before the tic occurs’), suggesting that the sensations of “itchiness” and “discomfort or uneasiness” do not tend to co-occur in the same individual. The negative correlation between PU1 and PU4 suggests that children may have different sensory characteristics (itch sensation dominant versus discomfort sensation or uneasy dominant). Clinically, behavioral interventions should be tailored to specific sensory experiences (e.g., habit reversal training) (17–19). For instance, distinguishing between tic behaviors that relieve ‘itchiness’ versus those that relieve ‘discomfort’ can help optimize treatment strategies. This is consistent with our team’s previous publication (12). However, while our previous research identified PU7 (‘These sensations often occur before the tic occurs’) as a critical node in the network, the current study identifies PU8 (‘These sensations almost always occur before the tic occurs’) as a critical node. The discrepancy may be due to two factors. First, this study used the Graphical Least Absolute Shrinkage and Selection Operator (GLASSO) algorithm to construct the network model, which was not used in the previous research. The advantage of this algorithm is that it removes of weak correlations, highlighting essential connections in the network structure, resulting in a clearer and more concise representation of the relationships between nodes. Secondly, PU7 and PU8 address similar content, both reflecting the transition from premonitory urge (PU) to tic occurrence. Therefore, whether PU7 or PU8 serves as a critical node, it implies that the transition from PU to tic is a crucial aspect of tic activation. These two items explore similar symptom dimensions, with PU8 focusing more on persistent premonitory urges, while PU7 emphasizes frequency. This change helps us better understand and implement behavioral therapies for clinical tic disorders Therefore, we speculate that Exposure and Response Prevention (ERP), by reducing the intensity of premonitory urges through habituation mechanisms (20, 21), may be more suitable for the characteristics of PU8; whereas Habit Reversal Training (HRT), by enhancing awareness of premonitory urges and training competing responses, may better align with the needs of PU7.

We focused our analysis on the PUTS-YGTSS network in order to gain a new perspective on the relationship between PU and tics. The historical debate in research on the relationship between PU and tics revolves around whether PU serves as a “driving force” behind tic generation or is a “by-product” of tics. Psychopathological network theory proposes that mental disorders are dynamic networks of interacting symptoms (17). Mental states are defined as dynamic equilibrium, and highly interconnected networks are more likely to develop into mental disorders. According to this theory, PU, together with motor tics, vocal tics, and tic impairment, form a dynamic component of ‘tic disorders’. Comparing the PUTS-YGTSS networks in the low and high age groups, we observed stronger connections in the low age group, consistent with previous research that tic disorders typically begin at 6–8 years of age. Psychopathological network theory also suggests the existence of a lag phenomenon in interconnected symptom networks, resulting in sustained network activation (22). This phenomenon is evident in the PUTS-YGTSS network. In tic disorder assessment interviews, some children express, ‘I used to blink because my eyes itched, but then it became a habit’. This phenomenon provides a nuanced explanation for the individual differences in the relationship between PU and tic symptoms, as found by researchers.

The PUTS-YGTSS network highlights links between different symptoms, known as ‘bridge symptoms’ (10), with YGTSS_D (impairment score) being the most prominent ‘bridge symptom’. The impairment score has six levels, corresponding to a range of 0–50 points. For example, if tics lead to panic attacks, periodic distress or significant changes in the family, frequent teasing or social avoidance, it should be considered a moderate impairment, scoring 30 points. This emphasizes the importance of focusing on the impact of tics on the child’s life, education and other aspects, rather than just the symptoms themselves in the clinical management of tics. This is consistent with the perspective presented in the European guidelines for tic disorders, where the assessment of whether tics interfere with daily life is a crucial criterion for deciding whether medication is warranted (23). Future research could further explore the impact of tics on self-esteem, family life, social interactions, and academic or occupational performance. Relevant scales, such as quality of life scales, can be used in studies of tic disorders.

In the PUTS-YGTSS network structure, we observe that in both the higher and lower age group, YGTSS_M1 (number), YGTSS_M3 (intensity) and YGTSS_M4 (complexity) are closely connected, with YGTSS_M1 (number) having the closest association with YGTSS_M4 (complexity). Conversely, YGTSS_M2 (frequency) and YGTSS_M5 (interference) show minimal associations with the other three items. This is an interesting finding. We know that the highest scores for number of movements and complexity are 5 points each. The criteria for the highest score for number involve “multiple discontinuous tics plus at least two instances of continuous or sequential complex tics, making it difficult to distinguish between discontinuous tics”. The criteria for the highest complexity score involve “long-duration, multiple muscle group tics that cannot be explained or disguised as reasonable normal behavior”. When continuous or sequential complex tics are present, “complexity” is at least moderate (involving multiple muscle groups simultaneously). This effectively explains the close relationship between YGTSS_M1 (number) and YGTSS_M4 (complexity). So, why do YGTSS_M2 (frequency) and YGTSS_M5 (interference) seem to be “separated” from the other three items? Does this mean that the frequency of motor tics and their interference with life are almost unaffected by the number, intensity, and complexity of motor tics? Clinical experience suggests that some children with tic disorders only exhibit the symptom of frequent eye blinking, which occurs almost continuously. However, as this symptom is relatively mild and the children have adapted to it, it does not significantly interfere with their learning and daily life. This further emphasizes the need to consider both the symptoms and their impact when dealing with children with tic disorders, rather than focusing on just one aspect.

By comparing the network characteristics between the different age groups, we observed a significant difference in the strength of the PU9 node (sensation of itching, tension, pressure, discomfort, unease, or an unfinished feeling that gradually disappears or lasts a short time after tics) in both the PUTS and PUTS-YGTSS networks. The PU9 node was stronger in the lower age group. This suggests that the activation of this node is more likely to propagate throughout the network in the lower age group. Conversely, controlling this node could potentially enhance overall network control (24). This provides empirical support for the behavioral therapy theory of tic disorders and suggests that behavioral therapy may be more useful in children of a younger age. The fundamental principle of behavioral therapy for tic disorders is that individuals experience relief or temporary alleviation of abnormal sensations by expressing tics when different types of premonitory urges (PUs) are perceived. When the reduction of abnormal sensations relies on the occurrence of tics, it reinforces the expression of tic symptoms (25). Whether through exposure and response prevention or habit reversal training, allowing the occurrence of PUs while preventing the manifestation of tics can lead to a gradual reduction in abnormal sensations, consequently alleviating tics (26). In both the PUTS and PUTS-YGTSS networks, the higher strength of PU9 in the lower age group indicates that this reinforcement process has a more substantial impact on the onset and development of tic disorders in children under 9 years old. The therapeutic effect of behavioral treatment may be more pronounced in this age group. When using HRT or ERP to treat PU9, we prioritize the younger age group.

### 4.1 Limitations

Firstly, this study uses an observational research design based on clinical data and reports baseline data for treatment-naive individuals with newly onset tic disorders. The network structure may be different in transient tic disorders, chronic tic disorders and Tourette’s syndrome, requiring further in-depth investigation.

Secondly, this study is a cross-sectional design, and no causal relationship between symptoms can be observed.

Thirdly, comorbidity is common in tic disorders. However, this study did not consider comorbid factors. While our focus was on tic symptoms and premonitory urges in the treatment-naive tic disorder population, comorbidities may still influence the overall network, especially after the onset of tic symptoms for three years. Future research should consider comorbid factors.

## 5 Conclusion

In conclusion, this study validates the internal structure of the PUTS scale across different age groups from a network perspective. Simultaneously, it reveals the characteristics of the PUTS-YGTSS network structure in a Chinese population aged 6–12 years with newly onset, treatment-naive tic disorders. By enhancing the understanding of premonitory urges and tics through a network approach, this study provides theoretical guidance and practical basis for targeted behavioral therapy.

## Data Availability

The raw data supporting the conclusions of this article will be made available by the authors, without undue reservation.
