# Supplementary figures and images for: Characteristics of premonitory urge and tic symptom in different age groups from a network perspective
Source: Front Psychiatry. 2025 Jun 3;16:1530911. doi: 10.3389/fpsyt.2025.1530911 (PMC12171955; doi:10.3389/fpsyt.2025.1530911)

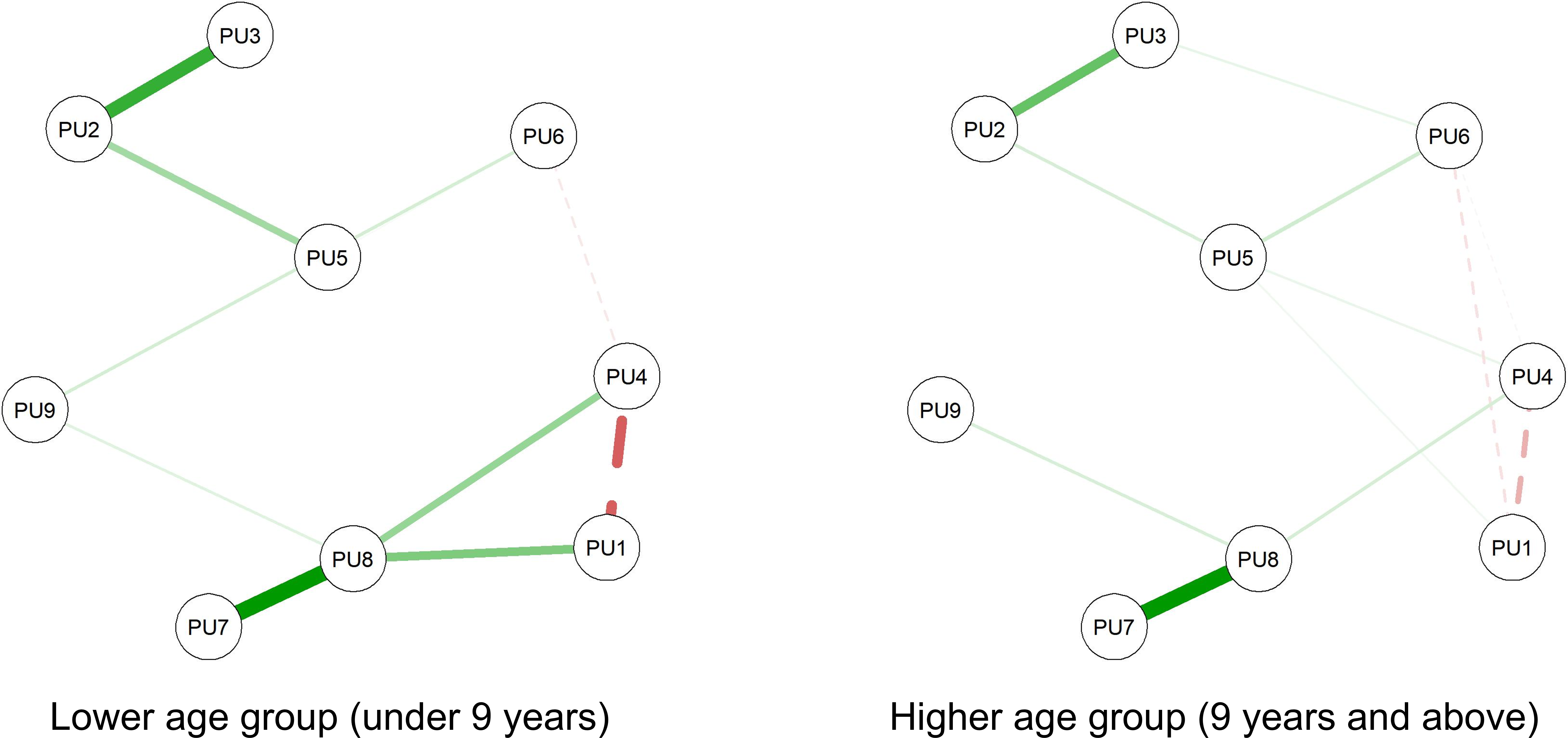

Supplement: Supplementary file 1 [file Image1.tif]

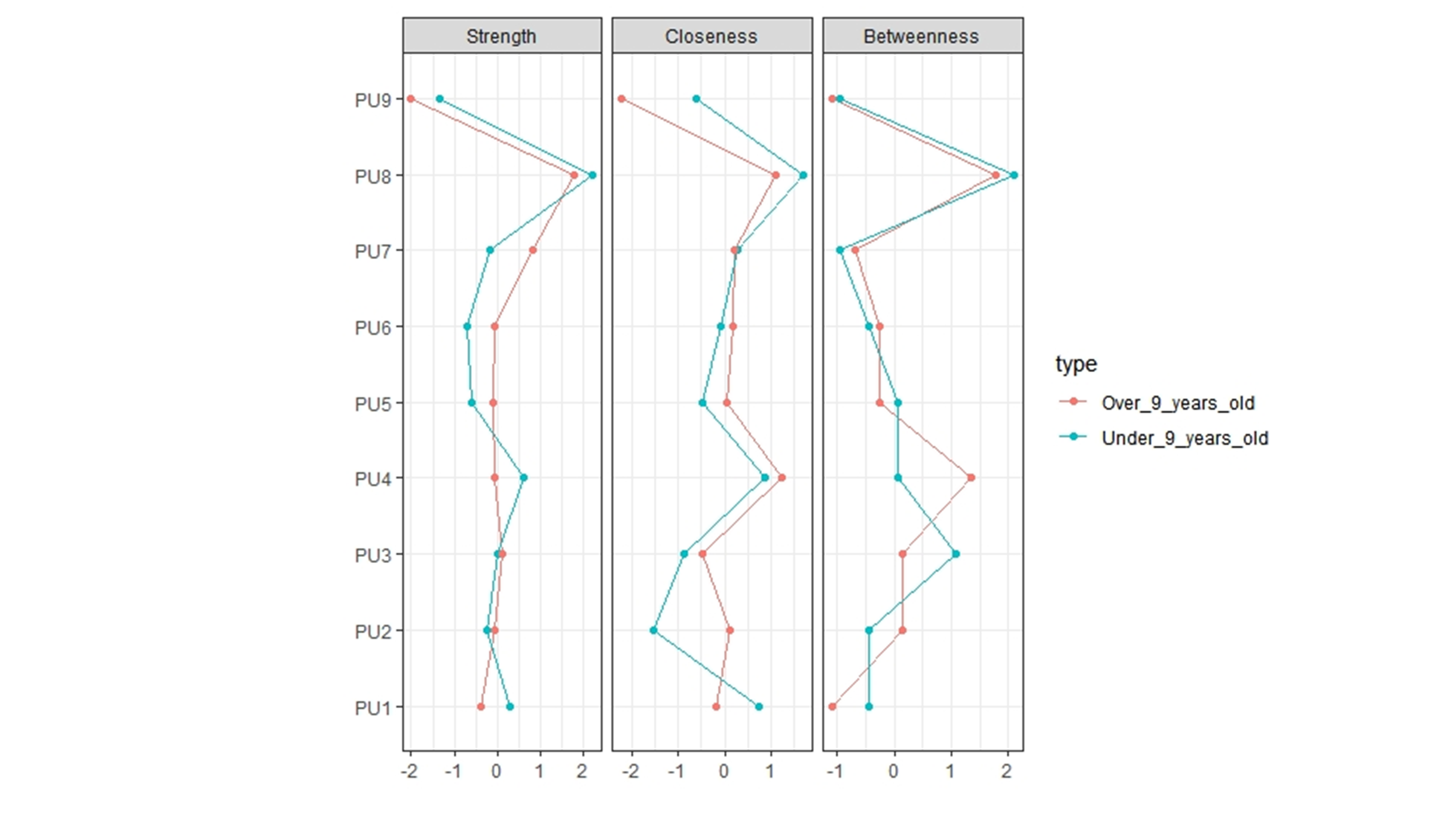

Supplement: Supplementary file 2 [file Image2.tif]

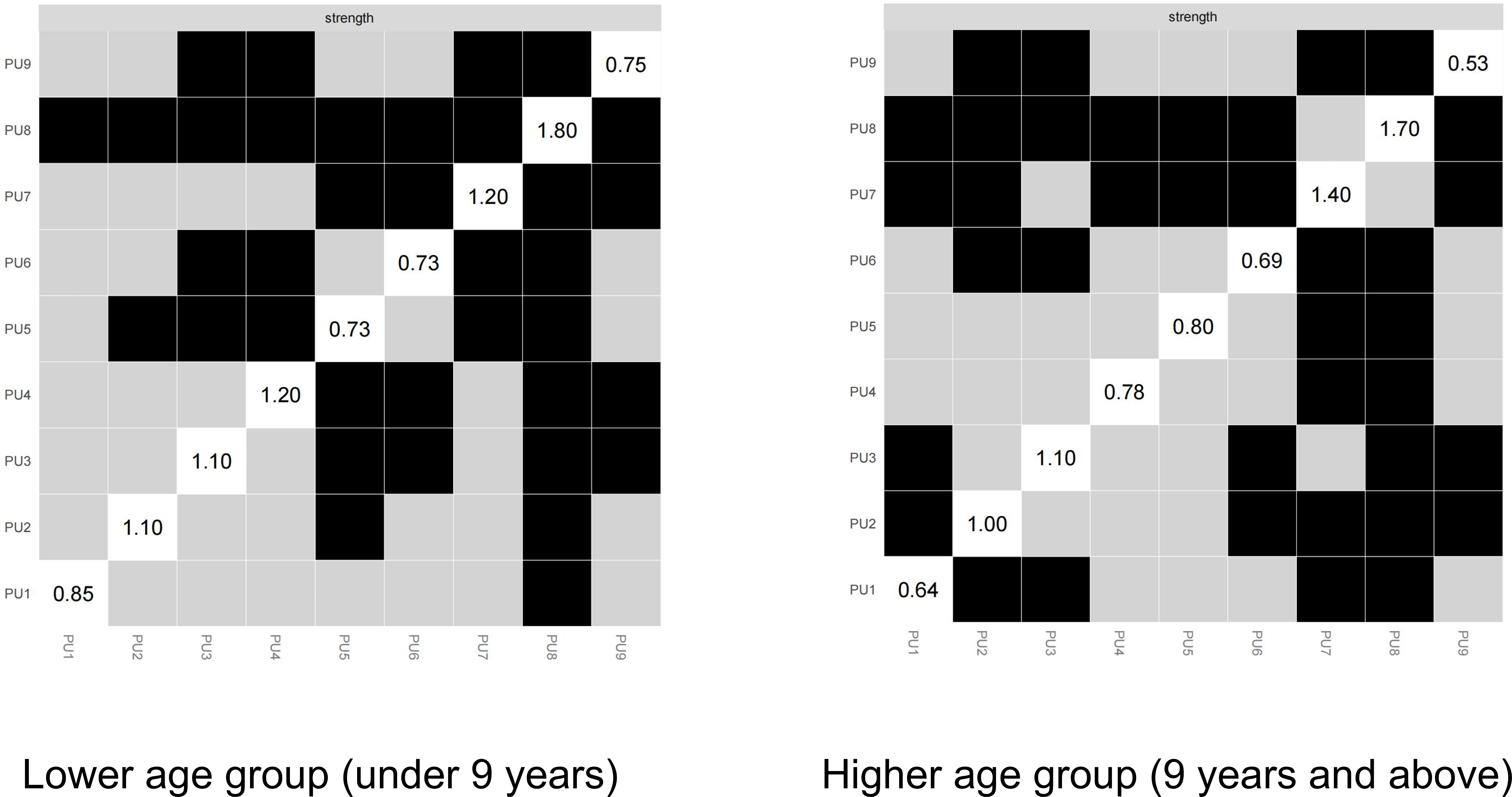

Supplement: Supplementary file 3 [file Image3.tif]

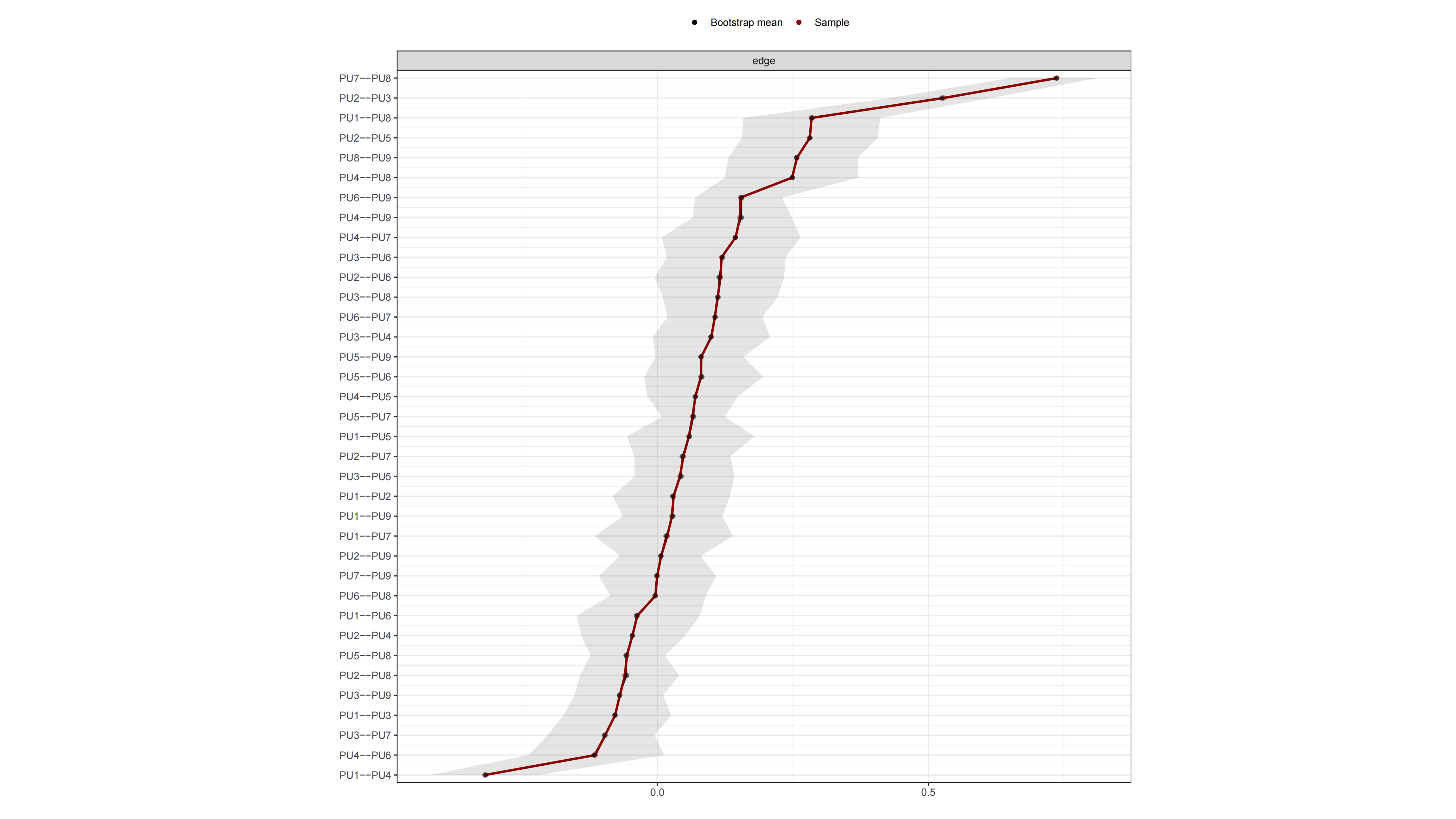

Supplement: Supplementary file 4 [file Image4.tif]
